# Synthesis of DMEA-Grafted Anion Exchange Membrane for Adsorptive Discharge of Methyl Orange from Wastewaters

**DOI:** 10.3390/membranes11030166

**Published:** 2021-02-27

**Authors:** Muhammad Imran Khan, Abdallah Shanableh, Javier Fernandez, Mushtaq Hussain Lashari, Shabnam Shahida, Suryyia Manzoor, Shagufta Zafar, Aziz ur Rehman, Noureddine Elboughdiri

**Affiliations:** 1 Research Institute of Sciences and Engineering, University of Sharjah, Sharjah 27272, United Arab Emirates; shanableh@sharjah.ac.ae; 2 Warwick Manufacturing Group, Coventry CV4 7AL, UK; j.fernandez@warwick.ac.uk; 3 Department of Zoology, The Islamia University of Bahawalpur, Bahawalpur 63100, Pakistan; mushtaqlashary@gmail.com; 4 Department of Chemistry, University of Poonch, Rawalakot 12350, Azad Kashmir, Pakistan; shabnamshahida01@gmail.com; 5 Institute of Chemical Sciences, Bahauddin Zakariya University, Multan 60800, Pakistan; suryyia.manzoor@bzu.edu.pk; 6 Department of Chemistry, The Government Sadiq College Women University, Bahawalpur 63000, Pakistan; shg_zf@gscwu.edu.pk; 7 Department of Chemistry, The Islamia University of Bahawalpur, Bahawalpur 63100, Pakistan; azizypk@yahoo.com; 8 Chemical Engineering Department, College of Engineering, University of Ha’il, P.O. Box 2440, Ha’il 81441, Saudi Arabia; ghilaninouri@yahoo.fr; 9 Chemical Engineering Process Department, National School of Engineering Gabes, University of Gabes, Gabes 6011, Tunisia

**Keywords:** DMEA, endothermic process, methyl orange, adsorption, anion exchange membrane, pseudo-second-order model

## Abstract

This manuscript describes the synthesis of dimethylethanolamine (DMEA)-grafted anion exchange membrane (AEM) by incorporating dimethylethanolamine as ion-exchange content into the polymer matrix via the solution casting method. The synthesis of the DMEA-grafted AEM was demonstrated by Fourier transform infrared (FTIR) spectroscopy. The prepared DMEA-grafted AEM exhibited higher thermal stability, homogeneous morphology, water uptake (W_R_) of 115%, and an ion exchange capacity (IEC) of 2.70 meq/g. It was used for the adsorptive removal of methyl orange (MO) from an aqueous solution via batch processing. The effect of several operating factors, including contact time, membrane dosage, initial concentration of aqueous dye solution, and temperature on the percentage discharge of MO and adsorption capacity, was evaluated. Experimental data for adsorption of MO onto the DMEA-grafted AEM was analyzed with two parameter and three parameter nonlinear adsorption isotherm models but fitted best using a nonlinear Freundlich isotherm. Adsorption kinetics were studied by using several models, and attained results showed that experimental data fitted well to pseudo-second-order kinetics. A thermodynamic study showed that adsorption of MO onto the prepared DMEA-grafted AEM was an endothermic process. Moreover, it was a feasible and spontaneous process.

## 1. Introduction

Several industries, including the plastics, textile, pharmaceutical, paper, and cosmetics industries, etc., widely utilize dyes and pigments to color their final products [1]. A crucial source of continuous environmental pollution is the textile dyeing process. It is associated with regularly enhancing the volume of wastewater possessing handled textile dyes. In the chemical industry, more than 7 × 10^5^ tons/year of dyes are utilized globally. Around 10% to 15% of them are expelled as drainage into water resources without any pretreatment. It is vigorously polluting the environment and poignant aquatic organisms as well as human life. There are more than 10,000 kinds of commercially available dyes, most of which are recognized as toxic for the environment. Moreover, several dye molecules can endure for a long time in the environment, and, resultantly, it is essential to discharge them from industrial drainage before their dumping in hydrological systems [2,3,4,5]. 

To date, a lot of methods, including ion exchange membrane (IEM) [6], reverse osmosis [7], flocculation [8], bacterial action, photocatalytic degradation [9], adsorption [10,11], etc. have been used for the exoneration of dyes and wastewater treatment. From these reported technologies, adsorption for wastewaters treatment is recognized as easy as pie due to its simple and low-cost method [12]. Moreover, it is an environment-friendly alternative due to room temperature and pressure and simultaneous discharge of dyes [13,14]. It gives an extraordinary surrogate for the treatment of wastewater, particularly when the adsorbent is reasonable and does not demand further pretreatment steps before its utilization [13,15].

In the past, researchers investigated the appropriateness of employing a lot of adsorbent with varying cost and efficiency such as waste apricot [16], coconut shells [17], dairy sludge [18], bamboo grass treated with concentrated sulfuric acid, peat [19], orange peels [20], peanut hulls [21], pistachio nut shells [22], rice husks [23], groundnut shell charcoal and bagasse [24], bamboo [25], jack fruit peels [26], date stones, and palm tree waste [27] for the discharge of heavy metals and dyes from wastewater. Currently, all the adsorbents used for the discharge of dyes and heavy metal ions depend on the interaction of the adsorbate with the functional groups of the adsorbents [28]. Thus, many adsorption sites and a large surface area of the matrix are crucial endowments for the adsorption capability of membranes to remove pollutants from wastewater [29,30,31]. Therefore an anion exchange membrane (AEM) can be recognized as a good option for the removal of dye from an aqueous solution due to its large surface area for adsorption. Ion-exchange membranes (IEMs) are one of the main technologies present in different energy and separation processes. Accordingly, IEMs are developed as a potential substitute adsorbent for industrial applications. Currently, many researchers are trying to find different cost-effective, sustainable substitutes for commercial activated carbon adsorbents. Moreover, it possesses cationic head groups, which are responsible for its excellent removal capacity of dye due to interaction with dye functional groups. Two kinds of membranes, including P81 and ICE450, were utilized for the adsorptive removal of methyl violet 2B aqueous solution [32]. In addition, Cibacron Blue 3GA was discharged from an aqueous solution by using AEMs [33]. Previously, we reported the applications of several commercial AEMs for the adsorption of dyes from aqueous solutions [13,34,35,36,37]. 

In this article, the synthesis of dimethylethanolamine (DMEA)-grafted AEM was carried out via the solution casting method by introducing DMEA as an ion-exchange moiety into the polymer matrix. The synthesis of DMEA-grafted AEM was accomplished by using Fourier transform infrared spectroscopy. The synthesized AEM was characterized in terms of water uptake, thermal stability, ion exchange capacity, and morphology. The prepared DMEA-grafted AEM was used for the adsorptive discharge of MO from an aqueous solution at room temperature. The effect of operational parameters on the percentage discharge of MO from aqueous solution and adsorption capacity was explored. Adsorption equilibrium, kinetics, and thermodynamics were also studied.

## 2. Experimental

### 2.1. Materials

Methyl orange, chlorobenzene, sodium chloride (NaCl), N-methyl-2-pyrrolidone (NMP), sodium sulfate (Na_2_SO_4_), dimethylethanolamine (DMEA), ethanol, potassium chromate (K_2_CrO_4_), chloroform, 2,2′-Azo-bis-isobutyro nitrile (AIBN), silver nitrate (AgNO_3_), and N-bromo-succinimide (NBS), were kindly supplied by Sinopharm Chemical reagent Co. Ltd, Shanghai, China and used as obtained. Throughout this research, deionized (DI) water was utilized. Poly (2,6-dimethyl-1,4-phenyleneoxide) (PPO) was kindly supplied by Sigma-Aldrich chemicals (Hamburg, Germany). 

### 2.2. Bromination of Poly(2,6-dimethyl-1,4-phenyleneoxide) (PPO)

Bromination of poly(2,6-dimethy-1,4-phenyleneoxide) (PPO) into brominated PPO (BPPO) was performed as described [38] (Figure S1) (See Section S1 for detail in supporting information). 

### 2.3. Synthesis of the DMEA-Grafted AEM

We employed the solution casting method to synthesize the homogeneous DMEA-grafted AEM, as reported in our previous work [39,40,41,42,43,44,45]. Initially, 0.8 g of BPPO was dissolved into 10 mL of NMP to get 8% (w%) homogeneous solution of it. The DMEA-grafted AEM was synthesized by introducing a measured amount (0.27 g) of DMEA into the casting solution. The reaction mixture was stirred at 40 °C for 16 hours and then cast onto a glass plate at 60 °C for 24 h. The DMEA-grafted AEM was peeled off the glass plate and cleaned with deionized water. Figure S1 depicts the chemical structure of the DMEA-grafted AEM. 

### 2.4. Characterization

#### 2.4.1. Instrumentations

Detailed instrumentation studies used the following instruments: ^1^H NMR (DMX 300 NMR spectrometer working at 300 MHZ), FTIR spectrometer (Vector 22, Bruker, Massachusetts, MA, USA), Field emission scanning electron microscope (FE-SEM, Sirion200, FEI Company, Hillsboro, OR, USA), and the Shimadzu TGA-50H analyzer (Kyoto, Japan) were used (See Section S2 for detail in supporting information).

#### 2.4.2. Measurement of Water Uptake and Ion Exchange Capacity of the DMEA-Grafted AEM

Water uptake of ion exchange membrane is a crucial factor. It has an important influence on the adsorption capacity of the ion exchange membranes. For the DMEA-grafted AEM, it was calculated by soaking the dried DMEA-grafted AEM into distilled water at an ambient temperature. The wet weight of the DMEA-grafted membrane was taken after discharge of surface water with absorbing paper. It was obtained from the difference in mass before and after drying the membranes using the following equation [46]

(1) $$W_{R}=\frac{W_{wer}-W_{dry}}{W_{dry}}\times 100\%$$

where *W_wet_* and *W_dry_* show wet and dry weights of the DMEA-grafted membrane respectively.

The classical Mohr’s method was employed to determine the ion exchange capacity of the DMEA-grafted AEM. Initially, the dried DMEA-grafted membrane was soaked in 1.0 M NaCl solution for 48 h to convert charge sites into the Cl^−^ form. It was then washed with deionized water to discharge excessive NaCl. After that, it was soaked in 0.5 M Na_2_SO_4_ solutions for 48 h. The quantity of Cl^−^ ions released was calculated by titration method by using 0.05 M AgNO_3_ as titrant and K_2_CrO_4_ as an indicator. It was estimated by the following equation

(2) $$IEC=\frac{C_{AgNO_{3}}V_{AgNO_{3}}}{m}$$

where *m*, *V*, and *C* depict the dry mass of the DMEA-grafted AEM, titer volume, and the concentration of AgNO_3_, respectively, used during titration.

### 2.5. Batch Adsorption Process

Herein, a batch adsorption procedure was used as described previously [13,14,34,35,36,47]. The solution of MO utilized was prepared by dissolving a determined quantity of methyl orange (MO) into distilled water at room temperature. The prepared DMEA-grafted AEM was shaken in a 20 mL MO solution at an agitation speed of 120 rpm. To find out the optimized contact time, the prepared DMEA-grafted AEM (the small pieces of DMEA-grafted AEM with a size of around 2 cm × 2cm) was shaken in 20 mL MO solution at an initial concentration of 50 mg/L at room temperature for different time intervals such as 100, 200, 300, 400, 500, 600, 900, and 1440 min. The optimized dosage of the DMEA-grafted AEM was determined by using the different amounts of the DMEA-grafted AEM such as 0.01, 0.02, 0.03, 0.04, and 0.05 g into 20 mL MO solution with an initial concentration of 50 mg/L of MO for 1440 min at room temperature. Adsorption isotherm study was carried out by shaking the prepared DMEA-grafted AEM (the small pieces of DMEA-grafted AEM with a size of around 2 cm × 2cm) (0.05 g) for 1440 min at room temperature into 20 mL MO solution with initial concentrations of 100, 200, 300, 400, 500, 800, and 1000 mg/L. To evaluate adsorption thermodynamics, a 20 mL MO solution with an initial concentration of 50 mg/L was utilized, and the membrane was shaken at 298, 313, 323, and 333 K for 1440 min with a constant membrane dosage of 0.05 g and stirred at a speed of 120 rpm. The UV/VIS spectrophotometer (UV-2550, SHIMADZU) was used to determine the concentration of MO by determining the absorbance of the supernatant at the wavelength λ_max._ = 464 nm for MO. The concentration of MO was determined from the calibration curve. The adsorption capacity and the percentage removal were determined by the below equations

(3) $$q_{t}=\frac{C_{0}-C_{t}}{W}\times V$$

(4) Removal=C0−CtC0×100

where *C_0_* and *C_t_* denote MO concentrations at the intital state at time *t*, respectively. Similarly, *V* indicates the volume of MO solution, and *W* is the weight of the prepared DMEA-grafted AEM, respectively.

### 2.6. Nonlinear Adsorption Isotherms

Several nonlinear adsorption isotherms were used to explain the adsorption of MO onto the DMEA-grafted AEM (See Section S3 for detail in supporting information).

### 2.7. Adsorption Kinetics

Adsorption kinetics was studied by using several kinetic models for MO adsorption onto the DMEA-grafted AEM (See Section S4 for detail in supporting information). 

### 2.8. Adsorption Thermodynamics 

Adsorption thermo dynamics were evaluated using the equations below

(5) $$\ln Kc=\frac{\Delta S^{o}}{R}-\frac{\Delta H^{o}}{RT}$$

(6) $$K_{c}=\frac{C_{a}}{C_{e}}$$

(7) $$\Delta G^{o}=\Delta H^{o}-T\Delta S^{o}$$

where *R*, ∆*G*^o^, *K*_c_, ∆*S*^o^, and ∆*H*^o^ are the general gas constant, change in Gibb’s free energy (KJ/mol), equilibrium constant, change in entropy (J/mol·K), and change in enthalpy (KJ/mol), respectively.

## 3. Results and Discussion

### 3.1. Bromination of Poly(2,6-Dimethyl-1,4-Phenylene Oxide)

The bromination of poly(2,6-dimethyl-1,4-phenylene oxide was attained by using AIBN as an initiator and NBS as a brominating agent. Based on reaction conditions and on the reagents utilized, bromination can occur either at the benzylic position or at the aromatic ring [38,45,48,49]. Herein, it took place at the benzylic position of PPO in the above-mentioned conditions in a refluxing chlorobenzene solution at 135 °C. To determine the structure and degree of bromination (DB) of BPPO, ^1^H NMR spectroscopy was used, and the attained ^1^H NMR spectrum is denoted in Figure S2. It indicated that the characteristic benzyl bromide group was at 4.3 ppm. The DB was 75%, as calculated from the integral area ratio between the unreacted benzyl signal at 2.1 ppm and the benzyl bromide group. 

### 3.2. FTIR and TGA Test

**Figure 1:**
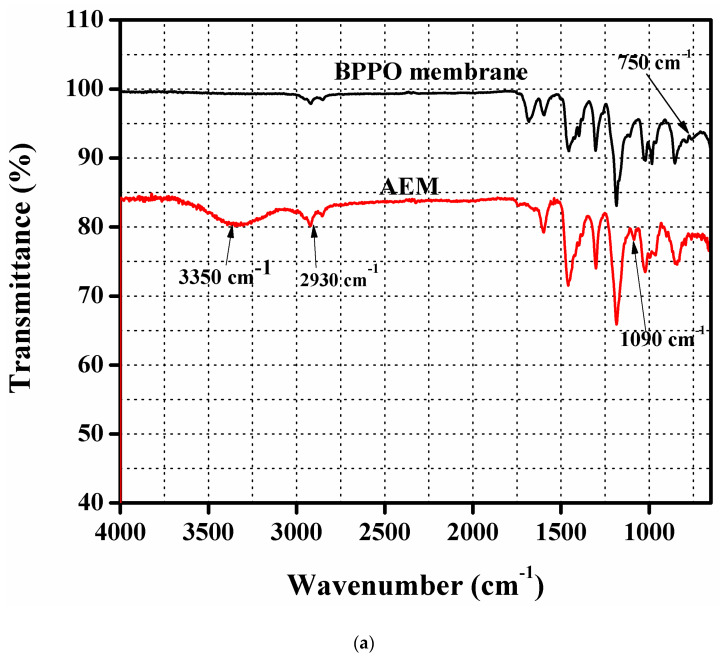

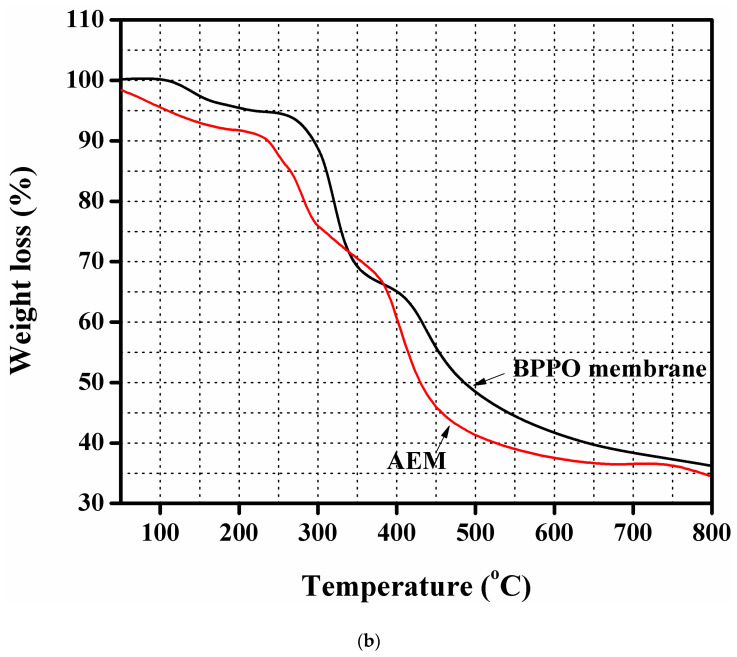
(**a**) IR spectrums of pure brominated of poly(2,6-dimethy-1,4-phenyleneoxide) (BPPO) as well as the dimethylethanolamine (DMEA)-grafted anion exchange membrane authenticating successful synthesis of the DMEA-grafted anion exchange membrane (AEM). (**b**) thermogravimetric analysis (TGA) thermograms of the pure BPPO membrane and DMEA-grafted AEM, indicating higher thermal stability of the DMEA-grafted AEM.

Figure 1a depicts FTIR spectrums of pure BPPO membrane as well as the prepared DMEA-grafted AEM. It was observed that a new peak appeared in the DMEA-grafted AEM at 1090 cm^−1^. It was because of the C-N stretching vibration into the spectrum of the DMEA-grafted AEM. It denoted the synthesis of the DMEA-grafted AEM, which was not present in the pure BPPO membrane. In the prepared DMEA-grafted AEM, the broad peak at 3350 cm^−1^ was due to the stretching vibration of an OH group. Moreover, the peak at 750 cm^−1^ was associated with the C-Br stretching vibration in the pure BPPO membrane [40]. It was not seen in the FTIR spectrum of the DMEA-grafted AEM [45,50]. The peak at 2930 cm^−1^ became broader because of the attachment of methyl groups (-CH_3_) containing DMEA to the polymer architecture [49]. It authenticated the successful synthesis of DMEA-grafted AEM. 

The thermal stability of the DMEA-grafted AEM was illustrated by thermogravimetric analysis (TGA). Figure 1b shows TGA thermograms of pure BPPO membrane as well as DMEA-grafted AEM. It was noted that the weight loss took place in three stages. Below 140 °C, the weight loss was due to the loss of solvent and residual water. In the polymer matrix, the degradation of a quaternary ammonium group was noted in the range of 180 to 240 °C, which corresponded to a second weight loss stage. The degradation of the polymer backbone at 460 °C led to the final weight loss stage. It demonstrated that the DMEA-grated AEM exhibited excellent thermal stability.

### 3.3. Morphological Study

The structure of ion exchange membranes also has a significant effect on the removal of dyes from an aqueous solution. It was investigated by scanning electron microscopy (SEM). Figure S3 depicts an SEM micrograph of the surface and cross-section of the synthesized DMEA-grafted AEM. There were no holes or cracks in the surface, and a cross-section of the prepared AEM demonstrated its homogeneous structure. But a small roughness was also noted in the cross-section of the membrane. Therefore, the prepared DMEA-grafted AEM was dense in nature and convenient for the adsorption of MO from an aqueous solution.

### 3.4. Water Uptake and Ion Exchange Capacity

Water uptake and ion exchange capacity indicate the hydrophilicity of the ion exchange membranes. These parameters are established on the amount of ion-exchange contents into a polymers architecture. For the prepared DMEA-grafted AEM, water uptake was found to be 115% at room temperature. Herein, ion exchange was estimated by employing classical Mohr’s method, and the obtained IEC value was found to be 2.70 meq/g. The theoretical IEC of the DMEA-grafted AEM was 2.85 meq/g. Water uptake and ion exchange capacity have a crucial effect on the adsorption efficiency of a prepared membrane. From this, we concluded that the synthesized membrane was highly hydrophilic and suitable for the adsorption of MO from an aqueous solution. 

### 3.5. Effect of Operating Factors on Adsorption of MO onto the DMEA-Grafted AEM

**Figure 2:**
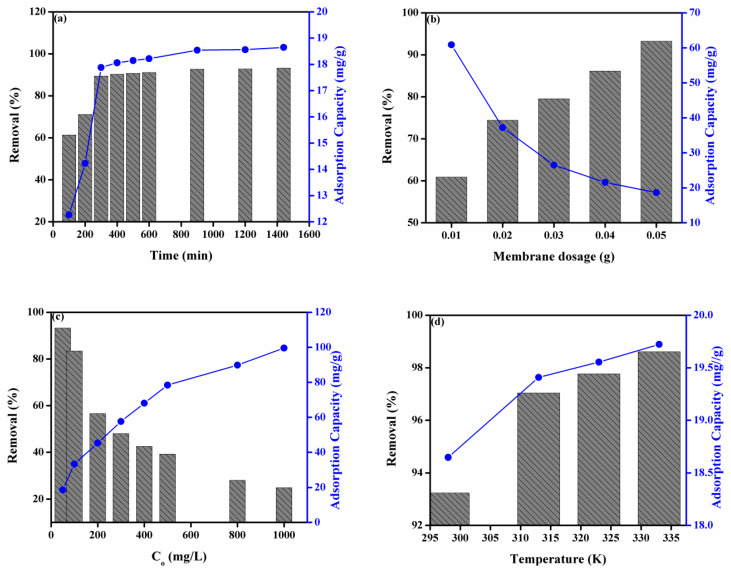
(**a**) Effect of contact time, (**b**) membrane dosage (adsorbent), (**c**) initial concentration of aqueous dye solution, (**d**) temperature on the percentage discharge of MO from aqueous solution and adsorption capacity.

Adsorption of dyes is dependent on operating endowments. In this manuscript, we investigated the influence of four operating parameters such as contact time, membrane dosage (the small pieces of DMEA-grafted AEM), initial concentration of dye solution, and temperature on the percentage discharge of MO from aqueous solution and adsorption capacity. Figure 2a represents the influence of contact time on the percentage discharge of MO and adsorption capacity. It was noted that the percentage discharge was increased from 61% to 93% and adsorption capacity from 12 to 19 mg/g with increasing contact time. It was observed that the enhancement in the percentage discharge of MO and adsorption capacity of the DMEA-grafted AEM was rapid in the initial step. The rapid removal of MO was due to the presence of many empty sites onto the DMEA-grafted AEM. After that, it slowed down over time due to the occupation of active sites onto the adsorbent (the DMEA-grafted AEM), and an equilibrium occurred after 900 minutes. Similarly, the adsorption capacity of the DMEA-grafted AEM was found to increase rapidly with enhanced contact time. Equilibrium was attained after 900 minutes, and this optimized time was used for further studies.

Figure 2b shows the effect of the membrane dosage on the percentage discharge of MO and adsorption capacity. It was observed that the percentage removal of MO was increased from 61% to 93% by increasing the membrane dosage from 0.01 to 0.05 g. Moreover, Figure S4 also depicts the influence of membrane dosage on adsorption of MO from aqueous solution onto the DMEA-grafted AEM at ambient temperature. It was due to the existence of a huge number of active sites with increasing the membrane dosage (adsorbent). Contrary, the adsorption capacity of the DMEA-grafted AEM was decreased by increasing the membrane dosage from 0.01 to 0.05 g. It was associated with the limited concentration of MO in the aqueous solution [51,52].

The effect of the initial concentration of dye solution onto the adsorption capacity of DMEA-grafted AEM and the percentage discharge of MO from aqueous solution is represented in Figure 2c. The percentage removal of MO from aqueous solution was decreased from 93% to 25 % by increasing the initial concentration of dye from 100 to 1000 mg/L. It was associated with the fact that the active sites of the DMEA-grafted AEM became saturated by increasing the initial concentration of dye, which resulted in a decrease in the percentage removal of dye from aqueous solution [13]. On the other hand, the adsorption capacity of the DMEA-grafted was increased from 19 to 100 mg/g by increasing the initial concentration of dye from 100 to 1000 mg/L. This was owed to the higher concentration of MO in the aqueous solution, which enhanced the migration of its molecules from the solution to the membrane surface. As a result, the interaction between MO molecules and the membrane surface was increased. Therefore, the initial concentration of dye had a positive effect on the adsorption capability of the prepared DMEA-grafted AEMs for MO.

Figure 2d represents the influence of temperature on the percentage discharge of MO and the adsorption capacity of the DMEA-grafted AEM for MO. It was observed that the percentage removal of MO from aqueous solution was increased from 93% to 99%, and the adsorption capacity of the DMEA-grafted AEM for MO also increased from 18.60% to 20% by increasing the temperature. It showed that the adsorption of MO from aqueous solution onto the prepared DMEA-grafted AEMs was an endothermic process.

### 3.6. Adsorption Isotherms

Two parameter nonlinear isotherms (Langmuir, Freundlich, Temkin, and Dubinin-Radushkevich (D-R)), and three parameter nonlinear isotherms (Redlich-Peterson (R-P), Hill, and SIPS) were also used to explain adsorption of MO onto the prepared DMEA-grafted AEM. All isotherm parameters were calculated by nonlinear regression by employing Igor Pro WaveMatrices 6.2.1 software [53]. The statistical tool needed for the best fit of adsorption data was a nonlinear chi-square test (χ2). A lower value represents similarities of the experimental results, while a higher value indicates a change in experimental results [54].

#### 3.6.1. Two Parameters Nonlinear Adsorption Isotherms

**Figure 3:**
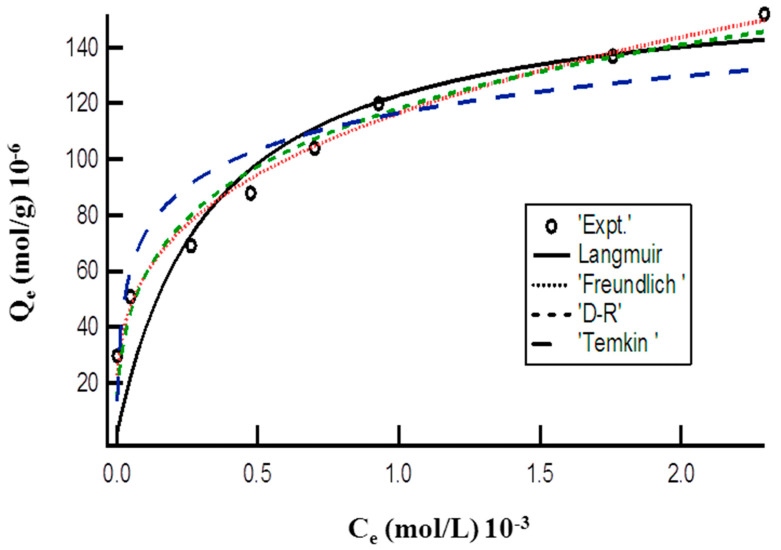
Plot of equilibrium concentration (C_e_) vs. adsorption capacity (Q_e_) representing nonlinear Langmuir, Freundlich, D-R, and Tempkin isotherms for adsorption of MO onto the DMEA-grafted AEM.

Figure 3 represents the plot of Langmuir, Freundlich, Dubinin-Radushkevich (D-R), and Temkin isotherm models for the adsorption of MO onto the DMEA-grafted AEM by a nonlinear method and attained the values of these isotherms parameter are given in Table 1. The attained lower values of the Chi-square showed that the experimental data for adsorption of MO onto the DMEA-grafted AEM followed the Langmuir, Freundlich, D-R, and Tempkin isotherm models, but best the fit was with the Freundlich isotherm model. The value of the Freundlich constant “n” shows favorability of the adsorption process, whereas K_f_ is the adsorption capacity of the prepared DMEA-grafted AEM. The attained value of mean adsorption energy (12.50 kJ/mol) from the D-R isotherm model demonstrated that adsorption of MO onto the DMEA-grafted AEM was a chemical ion-exchange adsorption process.

#### 3.6.2. Three Parameters Nonlinear Adsorption Isotherms 

**Figure 4:**
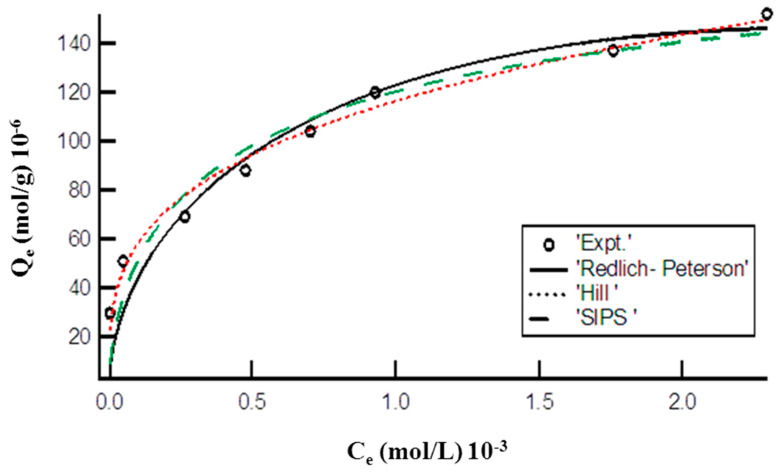
Plot of equilibrium concentration (C_e_) vs. adsorption capacity (Q_e_) representing nonlinear Hill, R-P, and SIPS isotherms for adsorption of MO onto the DMEA-grafted AEM.

Figure 4 represents the plot of Hill, R-P, and SIP models for the adsorption of MO onto the synthesized DMEA-grafted AEM. The calculated values of endowments of these applied three parameter isotherm models are given in Table 1. The lower value of chi-square showed that the adsorption of MO onto the prepared DMEA-grafted AEM could be defined by Hill, R-P model, and SIPS isotherm models. 

### 3.7. Adsorption Kinetics

**Figure 5:**
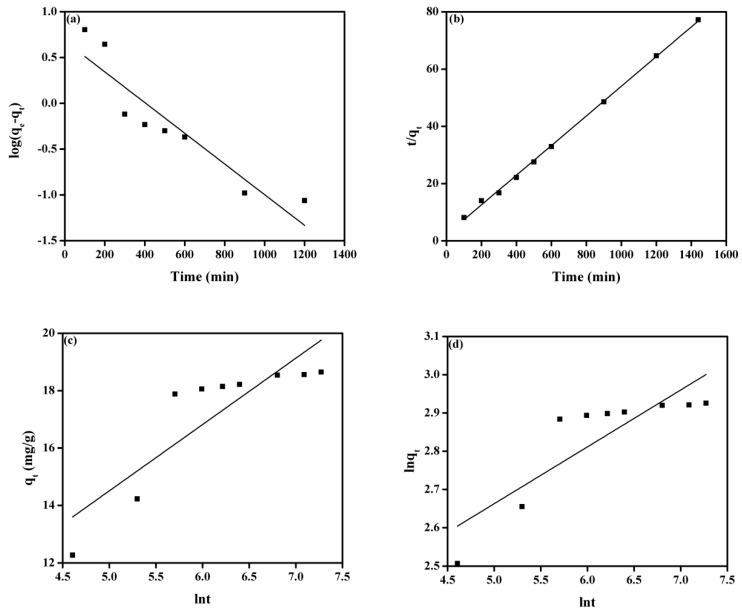
(**a**) Pseudo-first-order model, (**b**) pseudo-second model, (**c**) Elovich model, and (**d**) modified Freundlich equation for the adsorption of MO onto the DMEA-grafted AEM.

**Figure 6:**
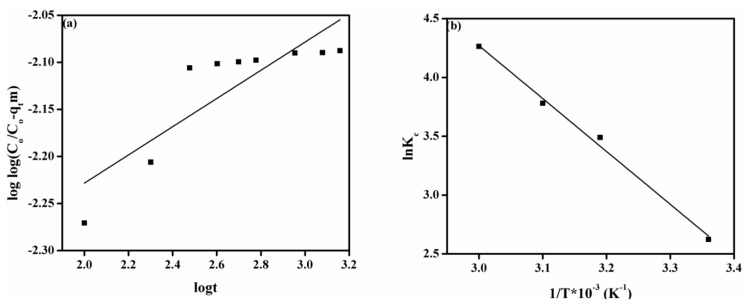
(**a**) Bangham model, (**b**) the plot of 1/T vs. lnK_c_ for adsorption of MO onto the DMEA-grafted AEM.

Figure 5a denotes the plot of pseudo-first-order model for adsorption of MO onto the DMEA-grated AEM, and attained values of parameters such as k_1_ and q_e_ are given in Table 2. The value of the correlation coefficient (R^2^) was 0.836. There were also large differences between experimental adsorption capacity (q_e_ and _exp._) and calculated adsorption capacity values (q_e_ and _cal._), indicating that a pseudo-first-order model can not explain the rate process. The plot of the pseudo-second-order model is shown in Figure 5b. From Table 2, it can be noted that the value of experimental adsorption capacity (18.65 mg/g) was close to the calculated adsorption capacity (19.61 mg/g). The value of the correlation coefficient (R^2^ > 0.999) was close to unity, demonstrating that the model fit the experiment data well. Similarly, the plots of the Elovich model and the modified Freundlich equation are shown in Figure 5c,d, respectively. As shown in Table 2, the value of values of correlation coefficient (R^2^) were 0.724 and 0.708 for the Elovich model and the modified Freundlich equation, respectively, show that these models were not suitable for explaining the experimental data. Moreover, the plot of the Bangham equation is represented in Figure 6a, and the calculated values of its endowment are given in Table 2. It did not provide a linear curve representing the diffusion of adsorbate (MO) into pores of the adsorbent (the DMEA-grafted AEM), showing it was not the only rate-limiting step [14] because both film and pore diffusion were crucial to different extents for the adsorption of MO from aqueous solution onto the DMEA-grafted AEM.

### 3.8. Adsorption Thermodynamics

Thermodynamical evaluation is crucial for the investigation of heat change, spontaneity, and feasibility of the adsorption process onto the surface of adsorbent and adsorption mechanism. Figure 6b indicates the plot of 1/T vs lnK_c_ for the adsorption of MO onto the prepared DMEA-grafted AEM, and the attained results of the thermodynamic study are given in Table 3. Results showed that the attained values of Gibbs free energy (Δ*G^o^*) were negative, demonstrating the spontaneity and feasibility of the MO adsorption process onto the DMEA-grafted AEM under the utilized experimental conditions. The positive value of enthalpy (Δ*H^o^*) confirmed that adsorption of MO onto the DMEA-grafted AEM was an endothermic process. Moreover, the positive value of entropy (Δ*S^o^*) exhibited the enhancement in randomness during adsorption of MO onto the DMEA-grafted AEM at the dye-membrane interface.

## 4. Conclusions

In summary, the DMEA-grafted AEM was successfully synthesized. The FTIR spectroscopy demonstrated the synthesis of the DMEA-grafted AEM. The synthesized AEM exhibited a homogeneous structure and excellent thermal stability. From an aqueous solution, the percentage MO removed was increased with membrane dosage, contact time, and temperature, whereas it declined by increasing the initial concentration of MO. Similarly, the adsorption capacity was increased with contact time, initial concentration of dye, and temperature, whereas it decreased with membrane dosage. The kinetic study represented that the adsorption of MO onto the DMEA-grafted AEM fit using a pseudo-second-order model. Isotherms evaluation indicated that adsorption of MO onto the grafted AEM was fitted well to the nonlinear Freundlich isotherm model. The thermodynamic adsorption study showed that adsorption of MO onto the DMEA-grafted membrane was a spontaneous and endothermic process. Hence, the synthesized DMEA-grafted AEM could be employed as an extraordinary candidate for the removal of MO from aqueous solution at an ambient temperature.

## Figures and Tables

**Table 1:** Measured isotherm parameters for MO adsorption onto the DMEA-grafted AEM by a nonlinear method.

Adsorption Isotherm	Parameters		χ2
Langmuir	Qm	1.63 × 10^−4^ ± 3.26 × 10^−6^	1.838 × 10^−9^
	KL	1.63 × 10^−4^	
Freundlich	KF	9.42 × 10^−4^	2.124 × 10^−10^
	n	3.3039	
D-R	Qs	2.98 × 10^−4^ ± 5.96 × 10^−6^	4.877 × 10^−10^
	β	3.22 × 10^−3^	
	E	12.50	
Temkin	bT	1.29 × 105	1.584 × 10^−9^
	aT	4.59 × 105	
Redlich-Peterson	KRP	0.090	1.033 × 10^−9^
	aR	0.014	
	β	0.61	
SIPS	Ks	0.025	8.998 × 10^−10^
	β	0.64	
	a	124	
Hill	qh	0.69	2.126 × 10^−10^
	nh	0.30	
	kd	733	

**Table 2:** Calculated values of kinetic equation rate constants represent that adsorption of MO followed pseudo-second-order model.

Kinetic Models	Parameters	Values
Pseudo-first-order model	Q _(exp.)_	18.65
	Q_e_	4.77
	k_1_	1.60 × 10^−3^
	R^2^	0.836
Pseudo-second-order model	q_e_	19.61
	k_2_	1.17 × 10^−3^
	R^2^	0.999
Elovich model	β	0.43
	α	8.35
	R^2^	0.724
Modified Freundlich equation	m	6.76
	k	0.136
	R^2^	0.708
Bangham equation	α	0.150
	k_o_	2.73 × 10^−3^
	R^2^	0.709

(k_1_: (/min); q_e_: mg/g; k_2_: g/mg.min; β: g/mg; α: mg/g.min; k: L/g.min; k_fd_: (/min); k_o_: mL/g/L.

**Table 3:** Calculated values of adsorption thermodynamic parameters indicating the adsorption of MO was a spontaneous and endothermic process.

Temperature (K)	ΔH	ΔS	ΔG
298			−43.92
313	37.40	147.50	−46.13
323			−47.61
333			−49.08

(ΔS: J/mol; (ΔH: KJ/mol; ΔG: KJ/mol)

## Supplementary Materials

The following are available online at https://www.mdpi.com/2077-0375/11/3/166/s1, Figure S1: Bromination of PPO and fabrication of DMEA-grafted anion exchange membrane., Figure S2: ^1^H NMR spectra of BPPO indicating successful bromination of PPO, Figure S3: SEM image of surface and cross-section of the grafted-anion exchange membrane representing homogeneous morphology. Figure S4: (a) MO aqueous solution before adsorption of methyl orange onto the DMEA-grafted AEM, (b) MO aqueous solution after adsorption methyl orange onto the DMEA-grafted AEM representing effect of membrane dosage on adsorption of MO dye from aqueous solution.

## Nomenclature

**Code**	**Full name**
AEM	Anion exchange membrane
BPPO	Brominated poly(2,6-dimethyl-1,4-phenylene oxide)
PPO	Poly(2,6-dimethyl-1,4-phenylene oxide)
MO	Methyl orange
