# Supplementary material for: Synthesis of DMEA-Grafted Anion Exchange Membrane for Adsorptive Discharge of Methyl Orange from Wastewaters
Source: Membranes (Basel). 2021 Feb 27;11(3):166. doi: 10.3390/membranes11030166 (PMC7997434; doi:10.3390/membranes11030166)
Supplement: Supplementary file 1 [file membranes-11-00166-s001.pdf]

membranes

Article

Synthesis of DMEA-Grafted Anion Exchange Membrane for Adsorptive Discharge of Methyl Orange from Wastewaters

Muhammad Imran Khan[1],* Abdallah Shanableh[1], Javier Fernandez[2], Mushtaq Hussain Lashari[3], Shabnam Shahida[4], Suryyia Manzoor[5], Shagufta Zafar[6], Aziz ur Rehman[7], Noureddine Elboughdiri[8,9]

[1] Research Institute of Sciences and Engineering, University of Sharjah, Sharjah 27272, United Arab Emirates; email: raoimranishaq@gmail.com, shanableh@sharjah.ac.ae
[2] WMG, University of Warwick, Coventry, CV4 7AL, UK; email: j.fernandez@warwick.ac.uk
[3] Department of Zoology, The Islamia University of Bahawalpur, Bahawalpur 63100, Pakistan; email: mushtaqlashary@gmail.com
[4] Department of Chemistry, University of Poonch, Rawalakot, Azad Kashmir, Pakistan; email: shabnamshahida01@gmail.com
[5] Institute of Chemical Sciences, Bahauddin Zakariya University, Multan 60800, Pakistan; email: suryyia.manzoor@bzu.edu.pk
[6] Department of Chemistry, The Government Sadiq College Women University, Bahawalpur 63000, Pakistan; email: shg_zf@gscwu.edu.pk
[7] Department of Chemistry, The Islamia University of Bahawalpur, Bahawalpur 63100, Pakistan; emails:azizypk@yahoo.com
[8] Chemical Engineering Department, College of Engineering, University of Ha'il, P.O. Box 2440, Ha'il 81441, Saudi Arabia
[9] Chemical Engineering Process Department, National School of Engineering Gabes, University of Gabes, Gabes 6011, Tunisia; E mail: ghilaninouri@yahoo.fr
* Corresponding authors: Tel: +971563404827; E-mail: raoimranishaq@gmail.com (M.I. Khan)

Section S1. Bromination of poly (2, 6-dimethyl-1, 4-phenyleneoxide) (PPO)

Typically, 5 g of poly (2, 6-dimethyl-1, 4-phenyleneoxide) (PPO) was dissolved into 50 mL of chlorobenzene in a round bottom flask containing a magnetic stirrer and reflexed condenser. NBS (7 g), and AIBN (0.25 g) were added and the solution was stirred at 135 oC for 3 hours. After cooling at 25 °C, the reaction mixture was poured into an excess of ethanol to precipitate the polymer. The polymer was filtered, washed with ethanol, re-dissolved into 60 mL chloroform and precipitated into excess of ethanol solution. The polymer was collected as a light-yellow powder and dried under vacuum for 2 days at 40 oC to attain BPPO with bromination ratio of 75 %.

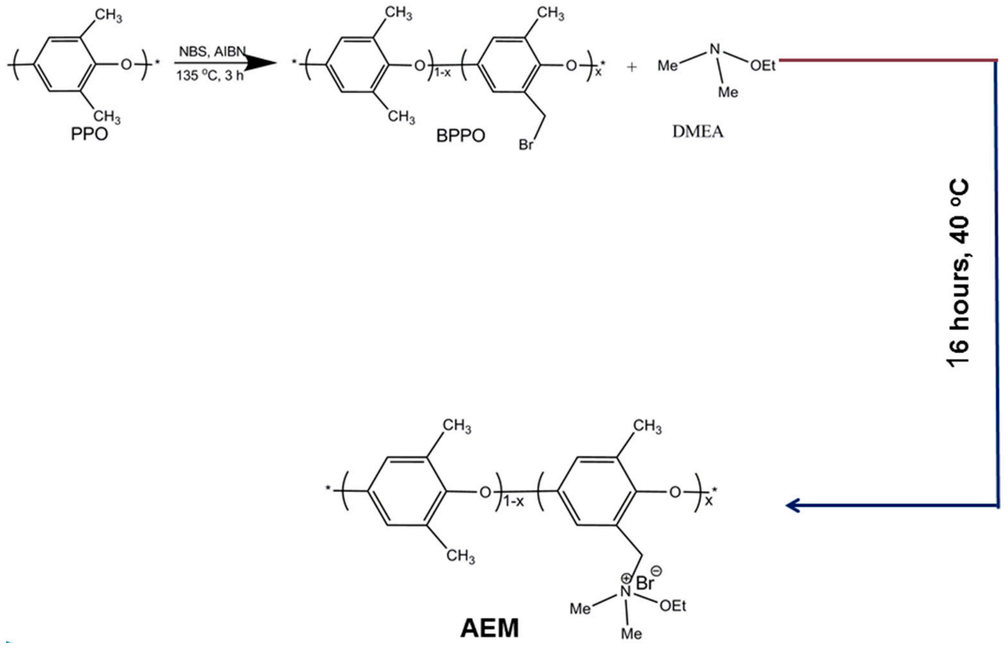

Figure 1. Bromination of PPO and fabrication of DMEA-grafted anion exchange membrane.

Section S2. Instrumentations

^{1}H NMR (DMX 300 NMR spectrometer operating at 300 MHZ) was utilized to confirm bromination of PPO. The successful fabrication of AEM was confirmed by using attenuated total reflectance (ATR) with FTIR spectrometer (Vector 22, Bruker, Massachusetts, MA, USA) in the range of 4000–400 cm^{-1}. Field emission scanning electron microscope (FE-SEM, Sirion200, FEI Company, Hillsboro, OR, USA) was employed to investigate morphology of the prepared AEM in detail. Similarly, the Shimadzu TGA-50H analyzer (Kyoto, Japan) under nitrogen flow with a heating rate of 10 ^{o}C/min within the temperature range of 25 to 800 ^{o}C was employed to study thermal stability of the developed AEM.

Section S3. Nonlinear adsorption isotherms

2.6.1. Two parameters adsorption isotherms

Herein, Langmuir, Freundlich, Dubinin-Radushkevich (D-R) and Temkin isotherms were employed to evaluate adsorption of MO onto the DMEA-grafted AEM.

Nonlinear Langmuir isotherm model is expressed as [10]

$$q_e = \frac{Q_m k_L C_e}{1+k_L C_e} \quad (1)$$

Copyright: © 2021 by the authors. Licensee MDPI, Basel, Switzerland. This article is an open access article distributed under the terms and conditions of the Creative Commons Attribution (CC BY) license (http://creativecommons.org/licenses/by/4.0/).

where K_L is Langmuir constant (L/mol) and Q_m is Langmuir monolayer adsorption capacity (mol/g).

Nonlinear Freundlich isotherm model is expressed as [10]

$$q_e = K_f {C_e}^{1/n} \quad (2)$$

where C_e is supernatant concentration at equilibrium state of the system (mol/L), and q_e is the amount of dye adsorbed at equilibrium state of system (mol/g), K_f and n are Freundlich parameters.

Dubinin-Redushkevich (D-R) model was utilized to distinguish between physical and chemical adsorption processes [10,11]. It is given by below equation

$$q_e = C_m \, exp\,(-\beta\varepsilon^2) \quad (3)$$

ε is the polanyi potential that is given as

$$\epsilon = RTln\left(1+\frac{1}{C_e}\right) \quad (4)$$

Where R is the universal gas constant (kJ/mol) and T is the absolute temperature (K). β is related to the mean adsorption energy by below relationship

$$E = \frac{1}{\sqrt{2\beta}} \quad (5)$$

Temkin isotherm is expressed as [10]

$$q_e = \frac{RT}{b_T}\ln(a_T C_e) \quad (6)$$

where b_T is related to the heat of adsorption and a_T is equilibrium binding constant coinciding to the maximum binding energy.

2.6.2. Three parameters adsorption isotherms

Experimental data for the adsorption of MO onto the prepared DMEA-grafted AEM was also subjected to three parameter isotherm models including, Hill, Redlich-Peterson and Sips which denote adsorption capacity as characteristic function of equilibrium concentration and are empirical in nature [10].

The nonlinear Hill adsorption isotherm is given as

$$q_e = \frac{q_H {C_e}^{n_H}}{k_H + {C_e}^{n_H}} \quad (7)$$

Redlich–Peterson isotherm contains elements of Langmuir and Freundlich isotherms which explain equilibrium on homogeneous and heterogeneous surfaces and multilayer adsorption. It possess three endowments such as a_{RP}, K_{RP} and g and is shown by as

$$q_e = \frac{K_{RP} C_e}{1 + a_{RP} {C_e}^g} \quad (8)$$

It is used to show adsorption equilibrium over a wide range of dye concentration molecules. The exponent g lies between 0 and 1. When β = 0, it becomes the Henry's law , and when β = 1, the R-P equation becomes the Langmuir equation [12].

SIPS isotherm was derived for determining the heterogeneous adsorption process and it is a combination of Langmuir and Freundlich isotherms [13]. Nonlinear SIPS adsorption isotherm is expressed by below equation:

$$q_e = \frac{K_S {C_e}^{\beta}}{1 + a_S {C_e}^{\beta}} \quad (9)$$

Section S4. Adsorption kinetics

2.7.1. Pseudo-first-order model

The Lagergren pseudo-first-order rate in linear form is represented as [1-3]

$$log(q_e - q_t) = \log q_e - \frac{K_1 t}{2.303} \quad (10)$$

where k_1 (/min), q_e and q_t are rate constant of pseudo-first-order model, concentration of MO adsorbed at equilibrium and time t respectively.

2.7.2. Pseudo-second-order model

The pseudo-second-order kinetic model linearized form is represented as [4-6]

$$\frac{t}{q_t} = \frac{1}{k_2 q_e^2} + \frac{t}{q_e} \quad (11)$$

where k_2 (g/mg.min) is the rate constant of pseudo-second-order model.

2.7.3. Elovich model

The Elovich model is expressed as [7,8]

$$q_t = \frac{1}{\beta}\ln(\alpha\beta) + \frac{1}{\beta}\ln t \quad (12)$$

where α (mg/g.min) and β (g/mg) are constant. The parameter α is initial adsorption rate and β is the extent of surface coverage and activation energy for chemisorption.

2.7.4. Modified Freundlich equation

It was originally developed by Kuo and Lotse [9,10]

$$q_t = kC_o t^{1/m} \quad (13)$$

where k, C_o, t and m are adsorption rate constant (L/g.min), initial concentration (mg/L), contact time (min) and the Kuo-Lotse constant respectively. Its linear form is shown as:

$$\ln q_t = \ln(kC_o) + \frac{1}{m}\ln t \quad (14)$$

3.7.5. Bangham equation

Bangham equation is shown as [9,10]

$$\log\log\left(\frac{C_o}{C_o - q_t m}\right) = \log\left(\frac{k_o m}{2.303V}\right) + \alpha \log t \quad (15)$$

where m is weight of the DMEA-grafted AEM utilized (g/L) ,V is volume of dye solution (mL), α (<1) and k_o (mL/(g/L) are constants.

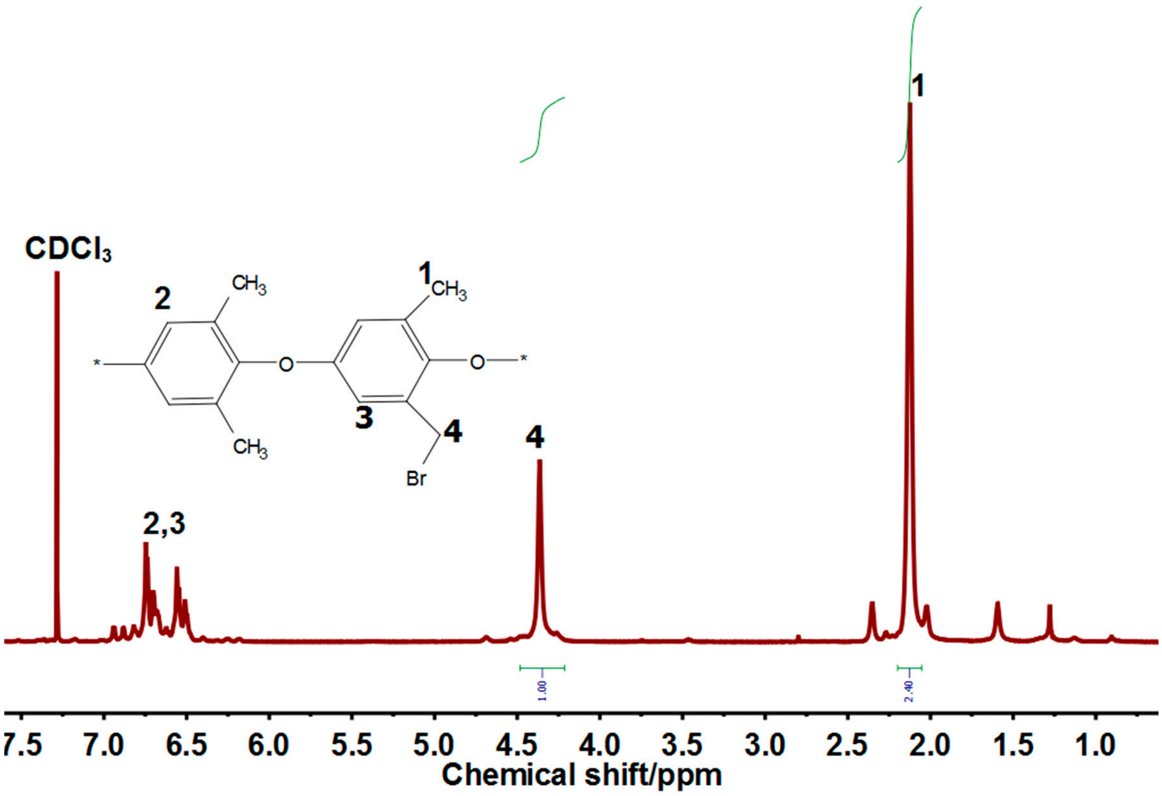

Figure 2. ^{1}H NMR spectra of BPPO indicating successful bromination of PPO.

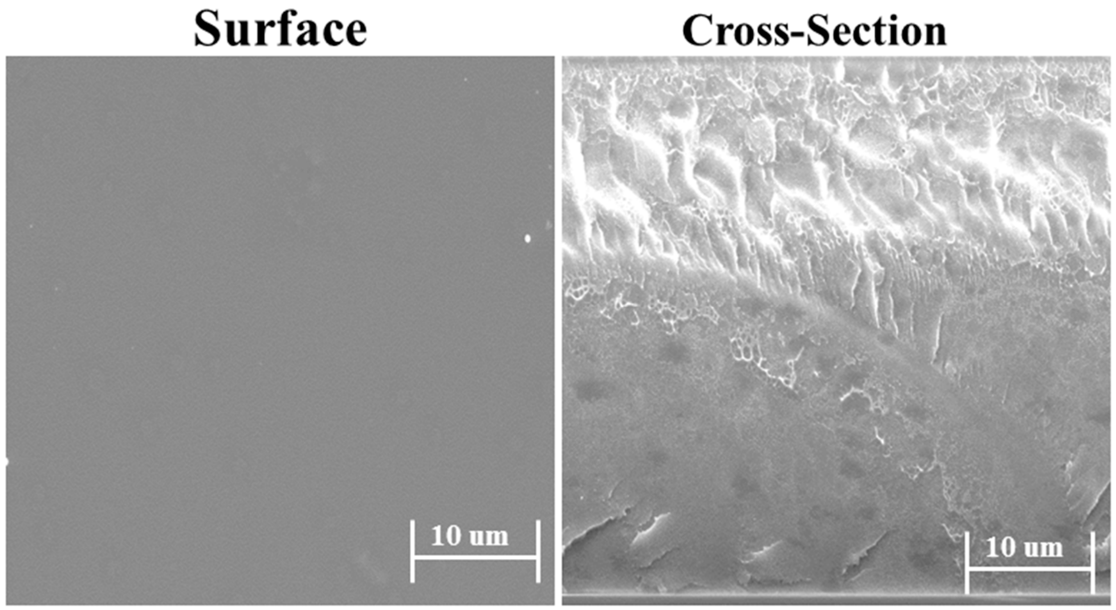

Figure 3. SEM image of surface and cross-section of the grafted-anion exchange membrane representing homogeneous morphology.

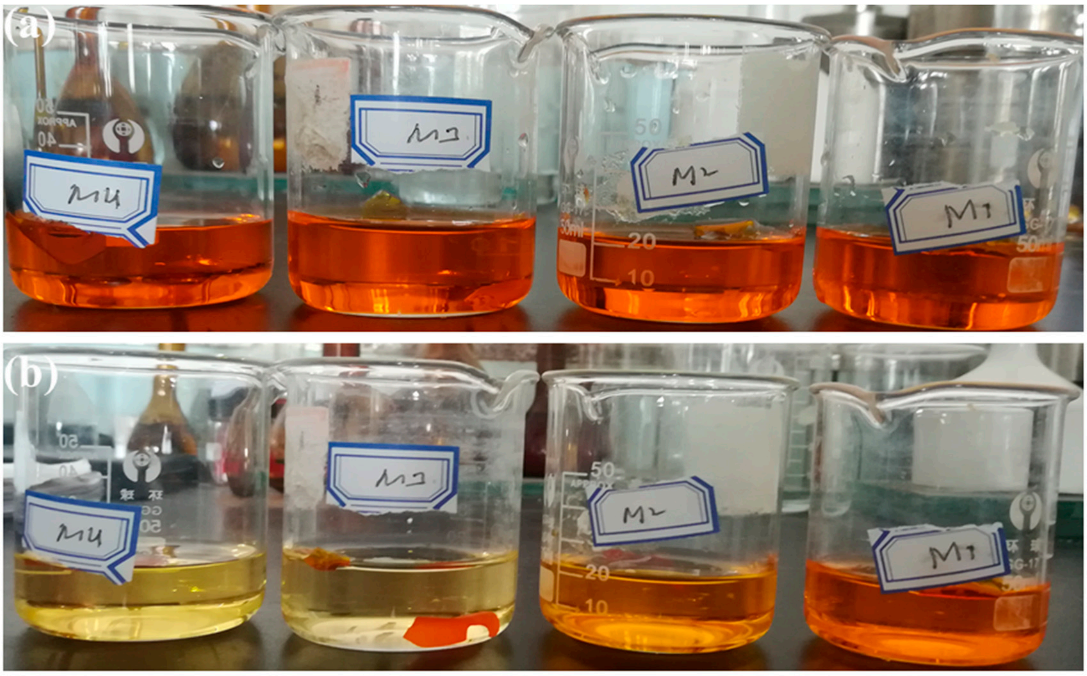

.

Figure 4. a) MO aqueous solution before adsorption of methyl orange onto the DMEA-grafted AEM, b) MO aqueous solution after adsorption methyl orange onto the DMEA-grafted AEM representing effect of membrane dosage on adsorption of MO dye from aqueous solution.
